# Phase‐Transition‐Promoted Interfacial Anchoring of Sulfide Solid Electrolyte Membranes for High‐Performance All‐Solid‐State Lithium Battery

**DOI:** 10.1002/advs.202407798

**Published:** 2024-10-22

**Authors:** Zhengkang Su, Qinzhe Zhou, Junhong Jin, Shenglin Yang, Guang Li, Jingjing Zhang

**Affiliations:** ^1^ State Key Laboratory for Modification of Chemical Fibers and Polymer Materials College of Materials Science and Engineering Donghua University Shanghai 201620 P. R. China; ^2^ Shanghai Aerospace Power Technology Co., LTD Shanghai 201112 P. R. China

**Keywords:** interfacial stability, ion‐conducting binder, solid‐to‐liquid·phase transition, solvent‐free manufacturing, sulfide solid‐state electrolyte

## Abstract

Solvent‐free manufacturing is crucial for fabricating high‐performance sulfide‐electrolyte‐based all‐solid‐state lithium batteries (ASSLBs), with advantages including side reaction inhibition, less contamination, and practical scalability. However, the fabricated sulfide electrolytes commonly suffer from brittleness, limited ion transport, and unsatisfactory interfacial stability due to the uncontrolled dispersion of the sulfide particles within the polymer binder matrix. Herein, a “solid‐to‐liquid” phase transition strategy is reported to fabricate flexible Li_6_PS_5_Cl (LPSCl) electrolytes. The polycaprolactone (PCL)‐based binder (PLI) with phase‐transition characteristics fills the gap of LPSCl particles and tightly grafts on the particle surface via ion‐dipole interaction, bringing a thin and compact electrolyte membrane (80 µm). The simultaneously high Li‐ion conducting and electron insulating nature of PLI binder facilitates Li‐ion transport and ensures good interfacial stability between electrolyte and anode. Consequently, the sulfide electrolyte membrane exhibits high ionic conductivity (8.5 × 10^−4^ S cm^−1^), enabling symmetric and full cells with 10 and 2.5 times longer cycling life compared with that of the cells with pristine LPSCl electrolyte, respectively. The demonstrated strategy is versatile and can be extended to ethylene vinyl acetate copolymer (EVA) that also brings enhanced electrochemical performance. The thin sulfide electrolyte with high interfacial stability potentially facilitates dendrite‐free ASSLBs with high energy density.

## Graphical abstract

The versatile “solid‐to‐liquid” phase transition strategy is proposed to fabricate thickness‐confined and structurally robust sulfide electrolyte membranes via a dry‐film process. The “liquid” polycaprolactone‐based Li‐conducting binder (PLI) fills the gap of sulfide electrolyte particles and tightly grafts on the particle surface via ion‐dipole interaction, bringing a compact electrolyte membrane with excellent ion transport capability and favorable interfacial compatibility.

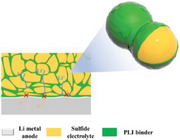

## 1 Introduction

Lithium‐ion batteries (LIBs) have been widely applied to power electric vehicles and portable electronics since their commercialization.^[^ ^1^ ^]^ However, the organic liquid electrolytes in conventional LIBs are flammable and prone to leakage, posing safety hazards in practical applications.^[^ ^2^ ^]^ In this regard, all‐solid‐state lithium batteries (ASSLBs) employing solid‐state electrolytes (SSEs) have been developed as promising alternatives to conventional liquid‐electrolyte‐based LIBs.^[^ ^3^ ^]^ The use of high‐voltage cathodes and high‐capacity Li metal anodes, both of which are compatible with SSEs, are also expected to endow ASSLBs with boosted energy density.^[^ ^4^ ^]^

Currently, sulfide electrolytes are considered one of the mainstream SSEs, exhibiting higher ionic conductivity compared with that of the polymer and oxide electrolytes and better ductility than oxide electrolytes that are inherently rigid.^[^ ^5^ ^]^ Owing to the high reactivity with water and organic solvents of the sulfides, the corresponding electrolyte membrane, which is essential to the Li‐ion transport and energy density of the full cell, is expected to be fabricated by a solvent‐free dry‐film technique.^[^ ^6^ ^]^ This poses a two‐fold challenge in selecting appropriate binder materials. The first is the limited among binders and electrolytes via solid‐solid contact constrain the dispersibility of the sulfides.^[^ ^7^ ^]^ This adds additional difficulties in constructing robust and thin solid‐state electrolyte membranes. Second, manipulating the dispersion of the sulfide particles within the binder matrix in a controlled and optimizable manner is crucial, yet remains difficult to use the dry‐film approach.^[^ ^8^ ^]^ Fully well‐dispersed sulfides in ionically isolating binder would disrupt the ion pathway,^[^ ^9^ ^]^ and incomplete encapsulation of the sulfide particles would also expose themselves in contact with the Li metal anode, leading to the formation of the interfacial passivation layer that potentially induces Li dendrite growth.^[^ ^10^ ^]^ The Li dendrite issue is further aggravated during the following repeated cycling due to the accumulation of local strain and uneven distribution of the electric field across the deteriorated interfacial passivation layer.^[^ ^11^ ^]^ Ultimately this would propagate the whole sulfide electrolyte until the ASSLBs experience a short circuit.^[^ ^12^ ^]^

A typical example of the dry‐film process is polytetrafluoroethylene (PTFE).^[^ ^13^ ^]^ Despite its wide applicability as a binder material,^[^ ^14^ ^]^ PTFE is unable to provide competent adhesion to disperse sulfides due to the limited intermolecular interactions.^[^ ^15^ ^]^ The inherent low stretchability and ionically isolating nature of PTFE also constrain the fabrication of thin layers of sulfides with robustness, flexibility, and favorable ionic conductivity.^[^ ^16^ ^]^ Furthermore, PTFE is readily converted to carbine‐type carbon via chemical/electrochemical reduction upon direct exposure to Li metal anodes.^[^ ^17^ ^]^ This carbine‐type carbon then rapidly yields electrically conducting *sp^2^* carbon deteriorating interfacial stability.^[^ ^18^ ^]^ Therefore, the development of advanced binder materials with competent interfacial adhesion, desirable ionic conductivity, and excellent chemical/electrochemical stability, as well as manufacturing practicability and scalability, is greatly desired to realize the full potential of the sulfide electrolytes for advanced ASSLBs.^[^ ^19^ ^]^

To improve the wettability and dispersibility of the sulfide particle within the binder matrix, we envision that it is feasible to introduce “liquid” into the dry‐film process by exploiting the solid‐to‐liquid phase transition of the binder. Enhanced flowability coupled with dispersibility can be achieved by melting the binder materials during the sulfide particle mixing. This also lays the foundations for accessing sulfide electrolytes/anode interface that conduct Li ions yet not electrons, which is ideal for suppressing Li dendrite formation. In this regard, we propose the fabrication of the Li_6_PS_5_Cl (LPSCl) solid electrolyte membranes using polycaprolactone (PCL)‐based Li‐conducting polymer binder (PLI) (labeled as LPSCl‐PLI) with outstanding ionic conductivity and ideal Li‐metal compatibility for advanced ASSLBs. The low melting point of PCL (80 °C) enables complete infiltration of the LPSCl particles with strong interparticle adhesions originating from the C═O and C─O─C groups in the molecular chain. Moreover, the LPSCl‐PLI electrolyte promotes smooth Li‐ion transport while inhibiting electron transport. The resulting improved interfacial compatibility between the PLI‐coated LPSCl electrolyte and the Li metal anode also substantially suppresses Li dendrite growth. Benefited from the favorable dispersibility, interfacial compatibility, and structural stability, the LPSCl‐PLI electrolyte with 7 wt% of PLI delivers a high room‐temperature ionic conductivity (8.5 × 10^−4^ S cm^−1^), an ultra‐stable cycling performance (1300 h) integrated in symmetric cells, and excellent electrochemical performances as assembled full cells. This strategy can be further extended to ethylene vinyl acetate copolymer (EVA), demonstrating its versatility to modulate the properties of polymer binders. Leveraging the phase transition of polymer binders at lower temperatures to prepare thin and robust sulfide SSE membranes poses a unique, effective, and versatile approach to resolving the dispersibility and compatibility issue, paving the way toward high‐performance sulfide‐electrolyte‐based ASSLBs.

## 2 Discussion

### 2.1 Synthesis and Characterization

**Figure 1:**
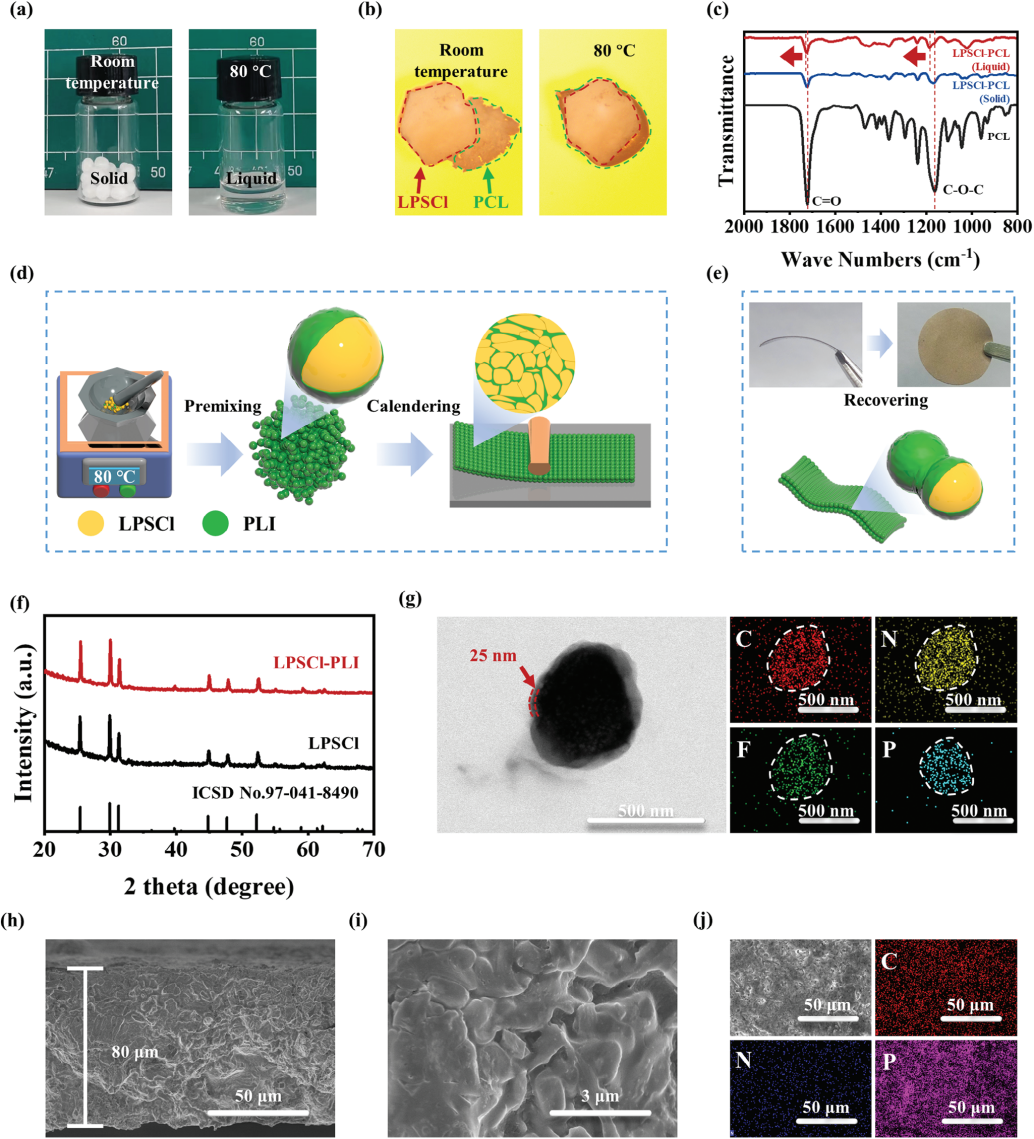
a) Digital images of PCL at room temperature and 80 °C. b) Optical images of PCL in contact with LPSCl electrolyte at room temperature and 80 °C. c) FTIR spectra of PCL, LPSCl‐PCL (liquid), and LPSCl‐PCL (solid). d) Illustration of the fabrication process of LPSCl‐PLI electrolyte. e) Bendability of LPSCl‐PLI electrolyte at room temperature. f) XRD patterns of LPSCl‐PLI and LPSCl electrolytes. g) TEM image of LPSCl‐PLI electrolyte and the corresponding STEM‐EDS elemental maps (C, N, F, and P). h,i) Cross‐sectional images of LPSCl‐PLI electrolyte. j) Top‐view scanning electron microscopy (SEM) image and corresponding EDS mapping images of LPSCl‐PLI electrolyte.

In principle, an ideal binder material for sulfide electrolytes should possess favorable dispersibility, good adhesion, high ionic conductivity, and excellent interfacial stability toward Li metal anodes.^[^ ^20^ ^]^ With these guidelines, PCL with lithium salt (lithium bis(trifluoromethanesulfonyl)imide, LiTFSI) and ionic liquid (N‐propyl‐N‐methylpyrrolidinium bis(trifluoromethanesulfonyl)imide, Pyr_13_TFSI) were explored. PCL readily achieves the solid‐to‐liquid conversion at 80 °C (**Figure** **1**a), which thoroughly mixes the LPSCl particles within the binder polymer matrix.^[^ ^21^ ^]^ As monitored in Figure 1b, heating up from room temperature to 80 °C induces uniform coating and dispersion of the sulfide electrolyte particles with the polymer binder in the liquid state. Fourier transform infrared spectroscopy (FTIR) spectroscopy was employed to probe the chemical bonding at the interface between LPSCl and PCL (Figure 1c). For the LPSCl‐PCL (liquid), a blueshift for the peaks of C═O and C─O─C (1726 and 1188 cm^−1^) attributed to PCL was observed.^[^ ^22^ ^]^ This undoubtedly demonstrates the interaction between these groups (C═O and C─O─C) and LPSCl, leading to strong adhesion among LPSCl particles.^[^ ^23^ ^]^ The LPSCl particles wrapped with PLI prevent degradation upon exposure to the Li metal anode, facilitating uniform Li plating.^[^ ^24^ ^]^ Notably, these interfacial interactions only occur when PCL is in the liquid state, which facilitates mixing and maximizes the interfacial contact. No significant peak shift was observed in the FTIR spectrum of the mixture of LPSCL and PCL both in the solid, and powder form. The Li‐ion conducting properties of PLI were also evaluated. The characteristic peaks at 1722 and 1162 cm^−1^ are attributed to the C═O and C─O─C groups in PCL observed in the FTIR spectra (Figure S1a, Supporting Information).^[^ ^25^ ^]^ For PLI, the peak at 1722 cm^−1^ splits into two peaks, implying coordination of the C═O group with Li ions for Li‐ion conduction.^[^ ^26^ ^]^ The incorporation of LiTFSI and Pyr_13_TFSI also decreases the crystallinity of PCL (Figure S1b, Supporting Information),^[^ ^27^ ^]^ leading to a room‐temperature ionic conductivity of 3.0 × 10^−5^ S cm^−1^ (Figure S2, Supporting Information).

The fabrication process of the LPSCl‐PLI membrane is schematically shown in Figure 1d. First, the LPSCl powder and PLI binder were premixed under 80 °C for 30 min, during which PLI transformed into a liquid to serve as a viscose glue to bind sulfide particles together. Then, the “dough” transforms into a thin film during the following calendering step. The as‐fabricated LPSCl‐PLI membrane showcases excellent flexibility and can be easily bent and recovered to flat after removing the external force (Figure 1e), achieving a small bending radius (5 mm, Figure S3, Supporting Information). As shown in Figure 1f, the powder X‐ray diffraction (XRD) pattern of LPSCl‐PLI resembles that of the commercial LPSCl (ICSD No. 97‐041‐8490), which indicates good chemical stability of PLI upon interfacing with LPSCl.^[^ ^9^ ^]^ The transmission electron microscopy (TEM) image confirms that the surface of the LPSCl particle is uniformly covered by the PLI binder (Figure 1g). In addition, scanning transmission electron microscopy‐energy dispersive spectrum (STEM‐EDS) elemental maps suggest C, N, and F derived from PLI span in a wider spatial range (≈500 nm) relative to that of P derived from LPSCl (≈450 nm), further confirming the formation of the PLI layer on the surface of LPSCl. X‐ray photoelectron spectroscopy (XPS) was conducted to unveil the surface chemical environments of PLI‐wrapped LPSCl electrolytes. A strong F 1s peak at 688.8 eV corresponding to the C‐F bond of the TFSI^−^ anion was observed for LPSCl‐PLI (Figure S4, Supporting Information), also demonstrating that PLI resides on the surface of the LPSCl particles.^[^ ^28^ ^]^ Moreover, this pre‐hot mixing approach delivers a much thinner LPSCl‐PLI membrane (80 µm) (Figure 1h) relative to the LPSCl membrane (450 µm) prepared by the cold pressing method (Figure S5, Supporting Information). Well‐connected LPSCl particles covered with PLI adhesive surfaces bring a compact electrolyte membrane with no obvious pores or cracks (Figure 1i). EDS elemental maps further reveal a homogeneous distribution of C, N (derived from PLI) and P elements (derived from LPSCl), implying uniform dispersion of LPSCl particles within the PLI network (Figure 1j).

**Figure 2:**
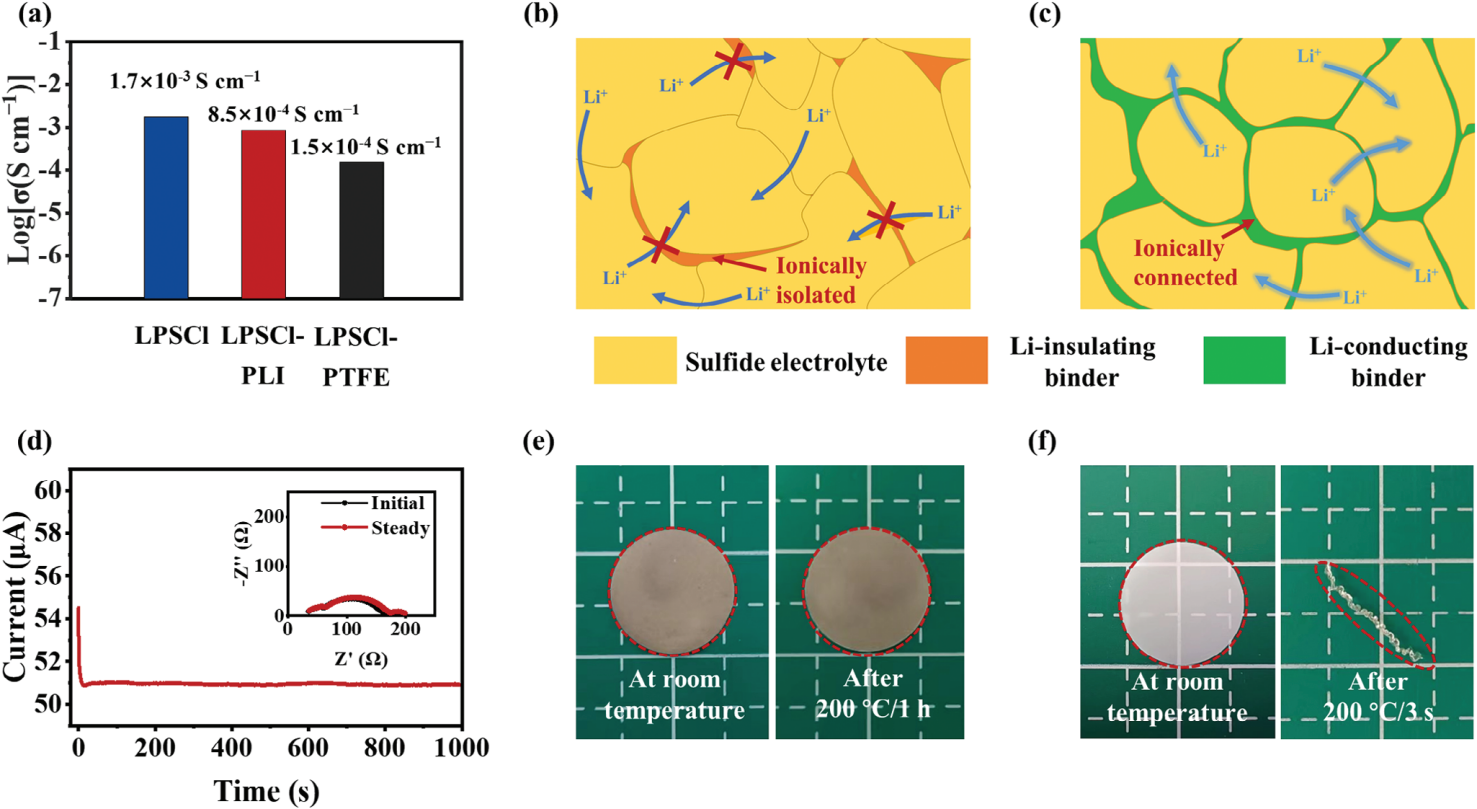
a) Ionic conductivities of LPSCl, LPSCl‐PLI and LPSCl‐PTFE electrolytes. Schematic diagram illustrating the microstructures of LPSCl electrolytes with b) Li‐insulating binder and c) Li‐conducting binder, and the highlighted blue arrows indicate Li ion pathways enabled by the Li‐conducting binder. d) Polarization curve of LPSCl‐PLI electrolyte (inset: initial and steady‐state Nyquist plots of the symmetric cell equipped with LPSCl‐PLI electrolyte at room temperature). Digital images of e) LPSCl‐PLI electrolyte and f) PP separator before and after thermal shrinkage at 200 °C.

The impact of binder materials on the ionic conductivities of sulfide electrolytes was interrogated using electrochemical impedance spectroscopy (EIS) measurement (Figure S6, Supporting Information). As summarized in **Figure** **2**a, the ionic conductivity of LPSCl‐PLI (8.5 × 10^−4^ S cm^−1^) is measurably higher than that of PTFE‐based LPSCl electrolyte (LPSCl‐PTFE) (1.5 × 10^−4^ S cm^−1^). Compared with pure LPSCl (1.7 × 10^−3^ S cm^−1^), the decreased ionic conductivity of LPSCl‐PTFE originates from the ionically‐insulating PTFE binder, which inevitably hinders Li‐ion transport in sulfide electrolytes (Figure 2b). This issue is alleviated by replacing PTFE with PLI, which brings excellent ion transport capability of LPSCl (Figure 2c). The Li transference number (t_Li+_) is another important factor in evaluating Li‐ion mobility. Figure 2d exhibits the polarization curve as well as the initial and steady‐state impedance diagram of the Li/LPSCl‐PLI/Li symmetric cell. The LPSCl‐PLI electrolyte exhibits a high t_Li+_ value (0.92), which effectively suppresses the space charge formation on the surface of Li metal anodes, thereby promoting smooth diffusion and uniform deposition of Li ions.^[^ ^29^ ^]^ The thermal stability of sulfide electrolytes was also evaluated. The LPSCl‐PLI membrane can maintain its original shape after heat treatment at 200 °C for 1 h (Figure 2e). Conversely, the PP separator melts when heated at 200 °C for 3 s (Figure 2f). These results indicate that the LPSCl‐PLI electrolyte with excellent thermal stability can remarkably improve the safety of Li metal batteries.

### 2.2 Electrochemical Performance

**Figure 3:**
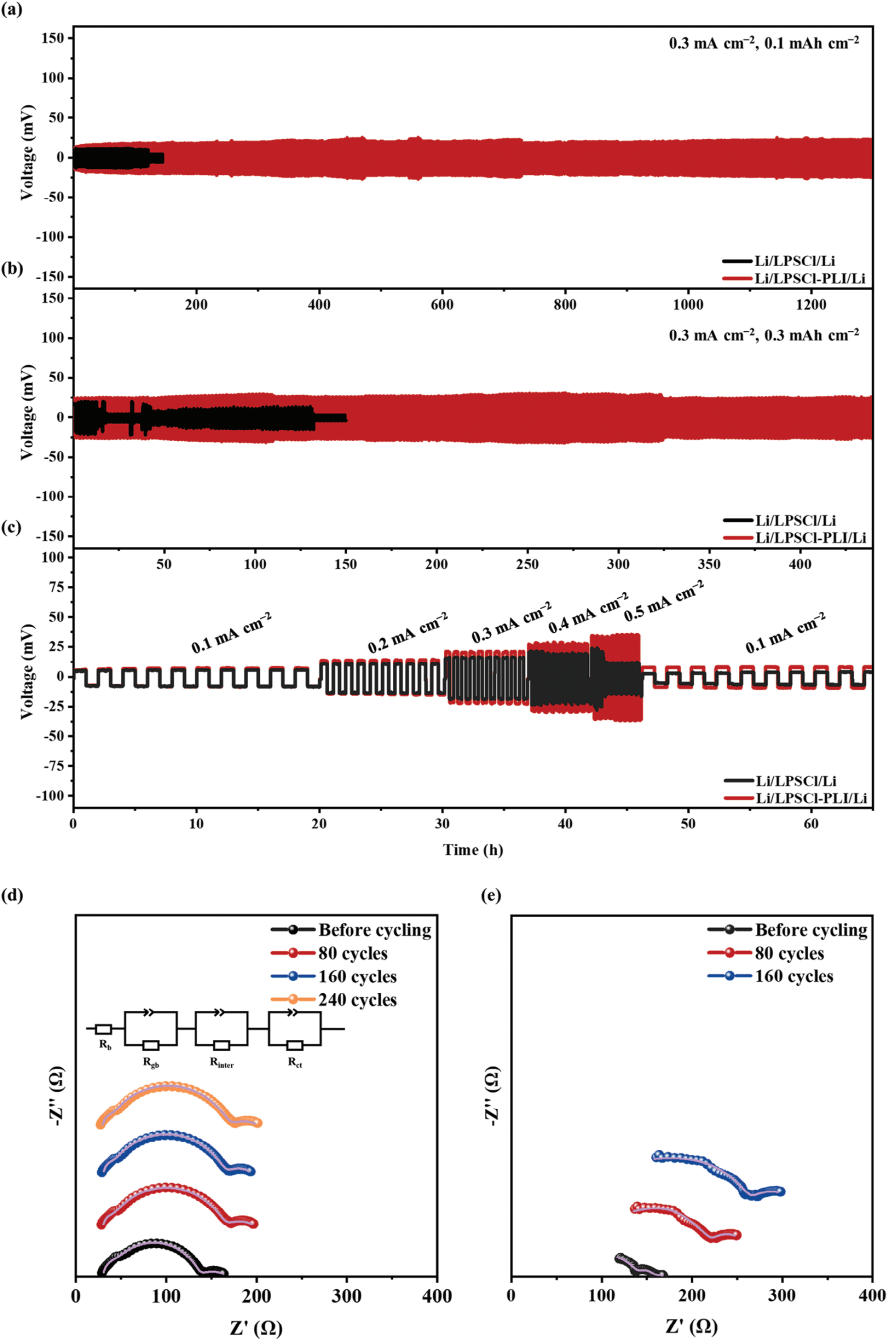
Comparison of cycling stability of Li/Li symmetric cells with LPSCl‐PLI and LPSCl electrolytes at a current density of 0.3 mA cm^−2^ at areal capacities of a) 0.1 and b) 0.3 mAh cm^−2^. c) Rate performances of Li/LPSCl‐PLI/Li and Li/LPSCl/Li symmetric cells at current densities ranging from 0.1 to 0.5 mA cm^−2^ at an areal capacity of 0.1 mAh cm^−2^. In‐situ EIS of Li/Li symmetric cells with d) LPSCl‐PLI (inset: the equivalent circuit for fitting the Nyquist plots) and e) LPSCl electrolytes during cycling.

The electrochemical stability of the solid electrolyte against the Li metal anode was assessed by operating Li symmetric cells under practical conditions (at room temperature, <1 MPa inherent to the coin cell).^[^ ^30^ ^]^ **Figures** **3**a and S7 (Supporting Information) show the voltage profiles of Li/LPSCl‐PLI/Li, Li/LPSCl/Li, and Li/LPSCl‐PTFE/Li symmetric cells at a current density of 0.3 mA cm^−2^ with an area capacity of 0.1 mAh cm^−2^. The Li/LPSCl‐PLI/Li cell exhibits stable Li plating/stripping performances up to 1300 h. For comparison, the Li/LPSCl/Li cell shows a short circuit at 126 h. Even worse, the Li/LPSCl‐PTFE/Li cell cannot survive in the first cycle due to the reaction between PTFE and Li metal anode. When the areal capacity is increased to 0.3 mAh cm^−2^ (Figure 3b), a small overpotential (26 mV) is also observed for the cell based on the LPSCl‐PLI electrolyte during the whole 440 h cycle, which is exceptional for sulfide SSEs (Figure S8 and Table S1, Supporting Information). In sharp contrast, the Li/LPSCl/Li cell presents voltage rise and fluctuation during cycling and shows severe polarization augment at 20 h. The compromised electrochemical performance of the Li/LPSCl/Li cell can be ascribed to the uncontrolled Li dendrite growth. Because of the large charge concentration gradient induced from the thick and nonuniform interfacial passivation layer on the Li metal anode, the interface deteriorates during the repeated cycling and thus leads to Li dendrite formation. Furthermore, compared to the LPSCl‐electrolyte‐based cell, the Li/LPSCl‐PLI/Li cell also exhibits improved rate performance at current densities ranging from 0.1 to 0.5 mA cm^−2^. A stable Li plating/stripping voltage curve is achieved at 0.5 mA cm^−2^ (Figure 3c), demonstrating the effectiveness of the LPSCl‐PLI electrolyte in improving the interfacial stability with the Li metal anode and suppressing Li dendrite formation.

To further understand the interface behaviors, in‐situ EIS measurements were carried out during cell cycling. An equivalent circuit was employed to analyze the impedance evolution of the bulk (R_b_), grain boundary (R_gb_), interface (R_inter_), and charge transfer (R_ct_) processes (Figure 3d,e). The fitting results are summarized in Figure S9 (Supporting Information). The R_b_ and R_gb_ are associated with the sulfide electrolyte in the cell. The Li/LPSCl‐PLI/Li cell exhibits observably lower R_b_ (29.6 Ω) and R_gb_ (7.7 Ω) values relative to that of the Li/LPSCl/Li cell. The R_b_ and R_gb_ values of the Li/LPSCl‐PLI/Li cell maintain unaltered during cycling. This indicates that the PLI binder residing on the surface of LPSCl particles prevents their continuous reaction with the Li metal anode, leading to excellent structural stability of LPSCl during the long‐term, repeated Li stripping/plating processes.^[^ ^31^ ^]^ In contrast, the R_gb_ value of the Li/LPSCl‐PLI/Li cell shows a continuous increase during cycling due to the occurrence of the side reaction. Additionally, the PLI brings a stable electrolyte/anode interface and a dendrite‐free Li deposition. The initial R_inter_ of the Li/LPSCl‐PLI/Li cell is 98.0 Ω, and slightly increases during the first 80 cycles due to passivation of the LPSCl‐PLI/Li interface. The R_inter_ gradually stabilizes during the following 240 cycles, indicating the dendrite‐free Li deposition is facilitated by the LPSCl‐PLI electrolyte. Although the initial R_inter_ of the Li/LPSCl/Li cell is smaller than that of the Li/LPSCl‐PLI/Li cell owing to the absence of PLI at the interface, an obvious increase in the R_inter_ and R_ct_ values is observed in the Li/LPSCl/Li cell during the whole 160 cycles. These results underscore the significant role of the PLI binder in suppressing the LPSCl/Li side reaction and stabilizing the passivation layer.^[^ ^32^ ^]^ To further probe the composition of the passivation layer, the XPS characterization was conducted. For the cycled Li metal anode from the Li/LPSCl‐PLI/Li cell, the peaks in the F1s and N 1s spectra at 688.8, 685.1, and 399.4 eV are attributed to the −CF bond, LiF and Li₃N, respectively (Figure S10a,b, Supporting Information), originating from the decomposition of TFSI⁻ in PLI binder.^[^ ^33^ ^]^ Additionally, the peaks corresponding to Li₂S and Li₃P are absent from the P 2p and S 2p spectra (Figure S10c,d, Supporting Information), indicating that the decomposition of LPSCl is suppressed. In contrast, the Li₂S and Li₃P peaks corresponding to Li₂S and Li₃P are observed on the Li metal anode surface from the cycled Li/LPSCl/Li cell (Figure S10e,f, Supporting Information).^[^ ^11^, ^34^ ^]^ The unregulated decomposition of LPSCl deteriorates the electrolyte/anode interface, thereby degrading battery performance.

**Figure 4:**
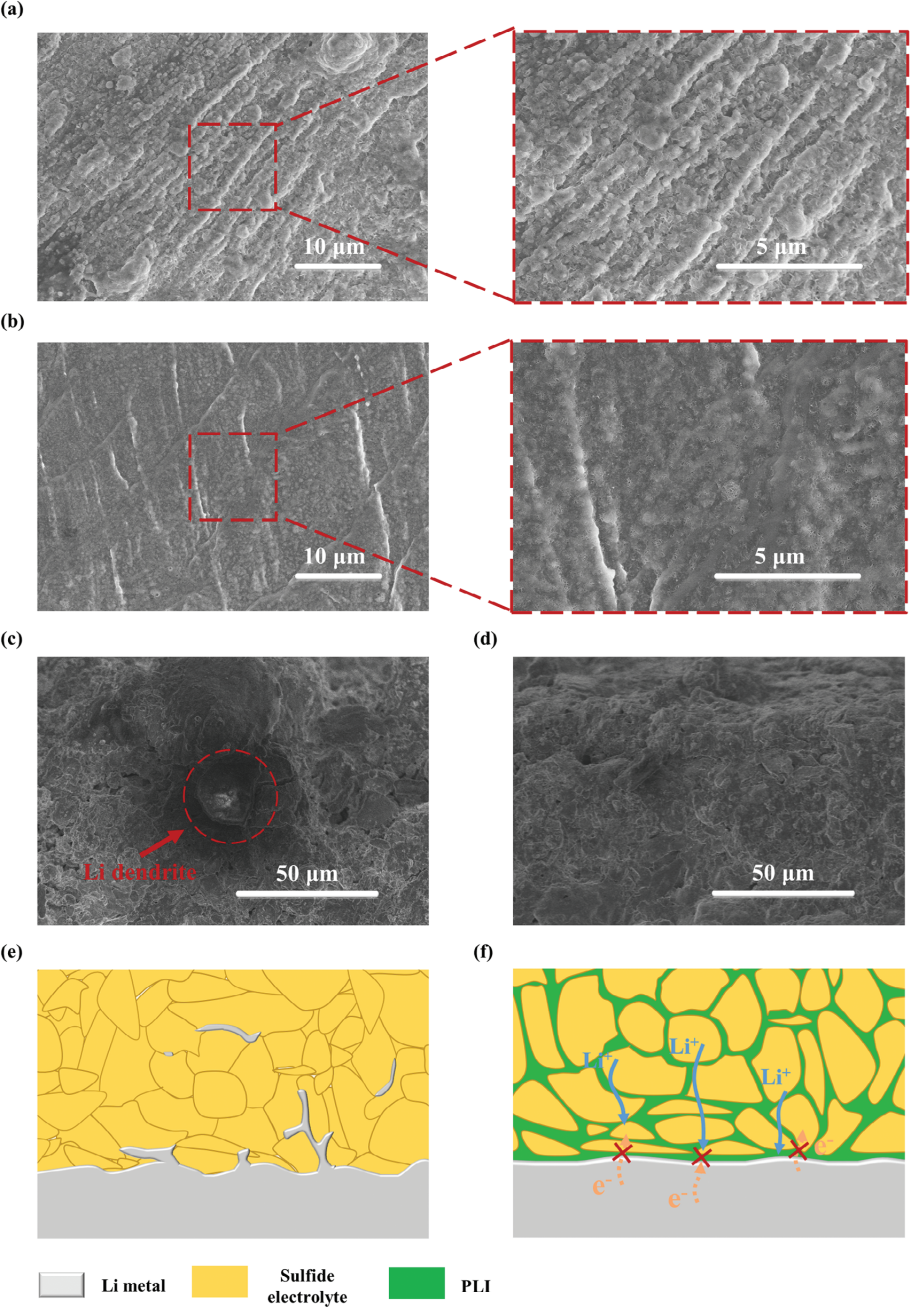
Top‐view SEM images of the Li metal anode assembled with a) LPSCl and b) LPSCl‐PLI electrolytes after 50 cycles at a current density of 0.3 mA cm^−2^. Cross‐sectional SEM images of c) LPSCl and d) LPSCl‐PLI electrolytes after 50 cycles at a current density of 0.3 mA cm^−2^. Schematic illustration of the different ionic/electronic conduction behaviors of e) LPSCl and f) LPSCl‐PLI electrolytes for Li dendrite suppression.

SEM characterization was conducted to compare the surface morphologies and structures of the symmetric cells using LPSCl‐PLI and LPSCl electrolytes. To alleviate the potential damage to the Li metal anode upon disassembling the symmetric cells, we applied an in‐situ stripping approach to remove LPSCl (Figure S11, Supporting Information).^[^ ^35^ ^]^ The emergence of accumulated Li deposits is shown on the surface of the Li metal anode from the Li/LPSCl/Li cell after 50 cycles, reflecting an unregulated Li plating/stripping process (**Figure** **4**a).^[^ ^36^ ^]^ Li deposition was also detected in bulk LPSCl electrolytes from the cross‐sectional SEM image (Figure 4c,e), which can be attributed to the continuous Li dendrite growth in electrolytes.^[^ ^37^ ^]^ By sharp contrast, the surface of the Li metal anode of the Li/LPSCl‐PLI/Li cell remains smooth without visible dendrites ascribed to the stabilized electrolyte/anode interface (Figure 4b).^[^ ^38^ ^]^ As a result, the growth of Li dendrites in the LPSCl‐PLI electrolyte is suppressed and the cycling life of the cells is prolonged (Figure 4d,f).

**Figure 5:**
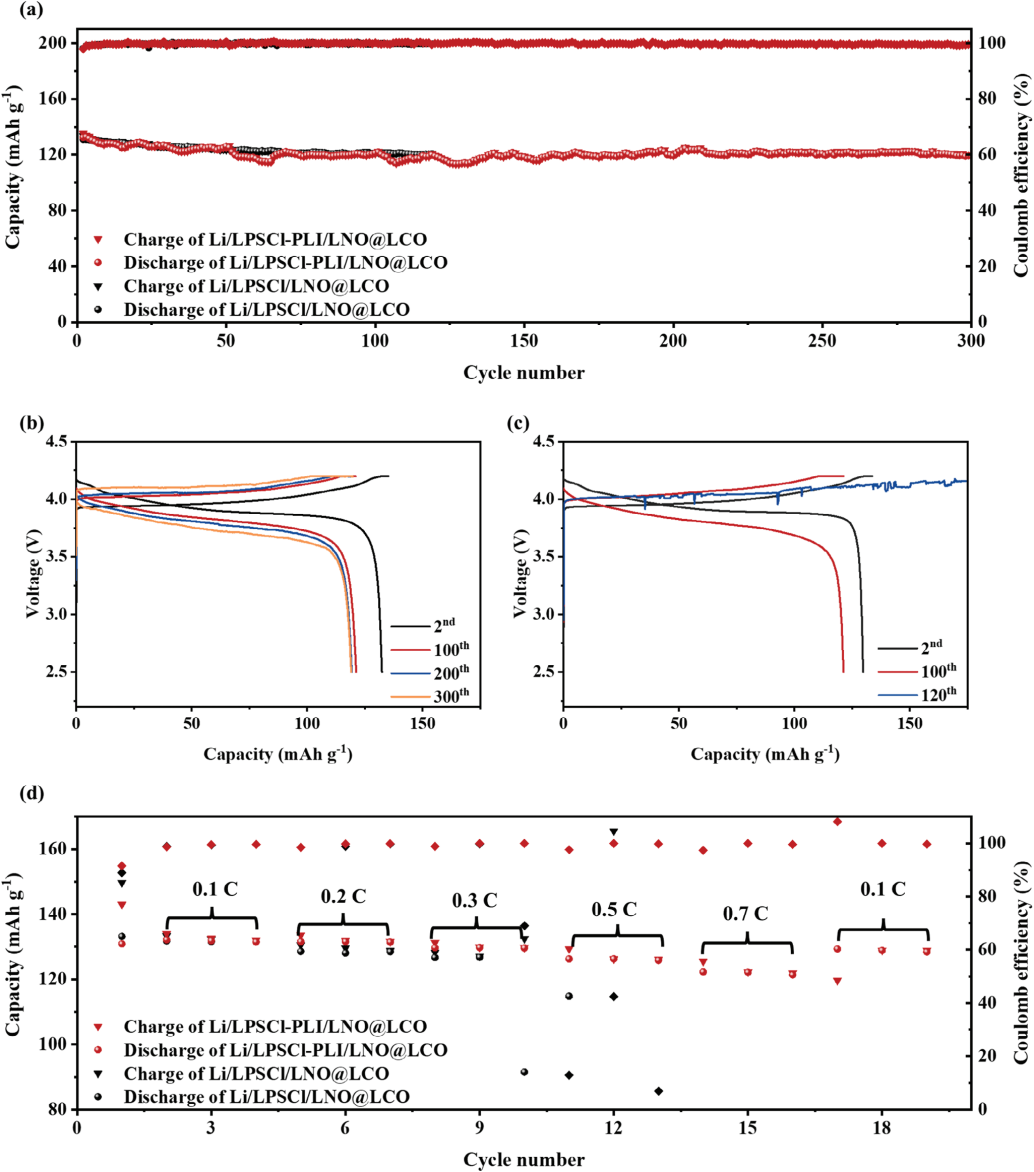
a) Cycling performances of Li/LCO cells assembled with LPSCl‐PLI and LPSCl electrolytes at 0.1 C and b,c) the corresponding voltage profiles at different cycles. d) Rate performances of Li/LCO cells assembled with LPSCl‐PLI and LPSCl electrolytes.

The LPSCl‐PLI electrolyte was further incorporated into full cells of ASSLBs, and LNO@LCO and Li foil is used as cathode and anode, respectively. The LCO particles are coated with LiNbO_3_ to prevent the interfacial side reactions between LCO and LPSCl.^[^ ^39^ ^]^ As shown in **Figure** **5**a,b, the Li/LPSCl‐PLI/LNO@LCO cell displays an initial reversible discharge capacity of 132.4 mAh g^−1^ after activation, and 119.0 mAh g^−1^ after 300 cycles, with an 89.9% capacity retention. It is noteworthy that the average Coulombic efficiency of the Li/LPSCl‐PLI/LNO@LCO cell is more than 99.7% during cycling, indicating highly reversible electrochemical reactions. In contrast, the formation of Li dendrites at the electrolyte/anode interface results in the cell short circuit of the Li/LPSCl/LNO@LCO cell,^[^ ^40^ ^]^ which exhibits a voltage drop at the 120^th^ cycle and fails to charge back (Figure 5a,c). The Li/LPSCl‐PLI/LNO@LCO cell also exhibits a better rate capability than that of the Li/LPSCl/LNO@LCO one (Figure 5d). The Li/LPSCl‐PLI/LNO@LCO cell exhibits reversible capacities of 132.2, 131.6, 129.7, 126.3, and 122.3 mAh g^−1^ at 0.1, 0.2, 0.3, 0.5, and 0.7 C, respectively. The cell delivers capacity retention of 99.5%, 98.1%, 95.5%, and 92.5% at 0.2, 0.3, 0.5, and 0.7 C, respectively, demonstrating excellent rate performance.^[^ ^34^, ^41^ ^]^ On the contrary, the Li/LPSCl/LNO@LCO cell short‐circuits at 0.3 C due to Li dendrite growth.

**Figure 6:**
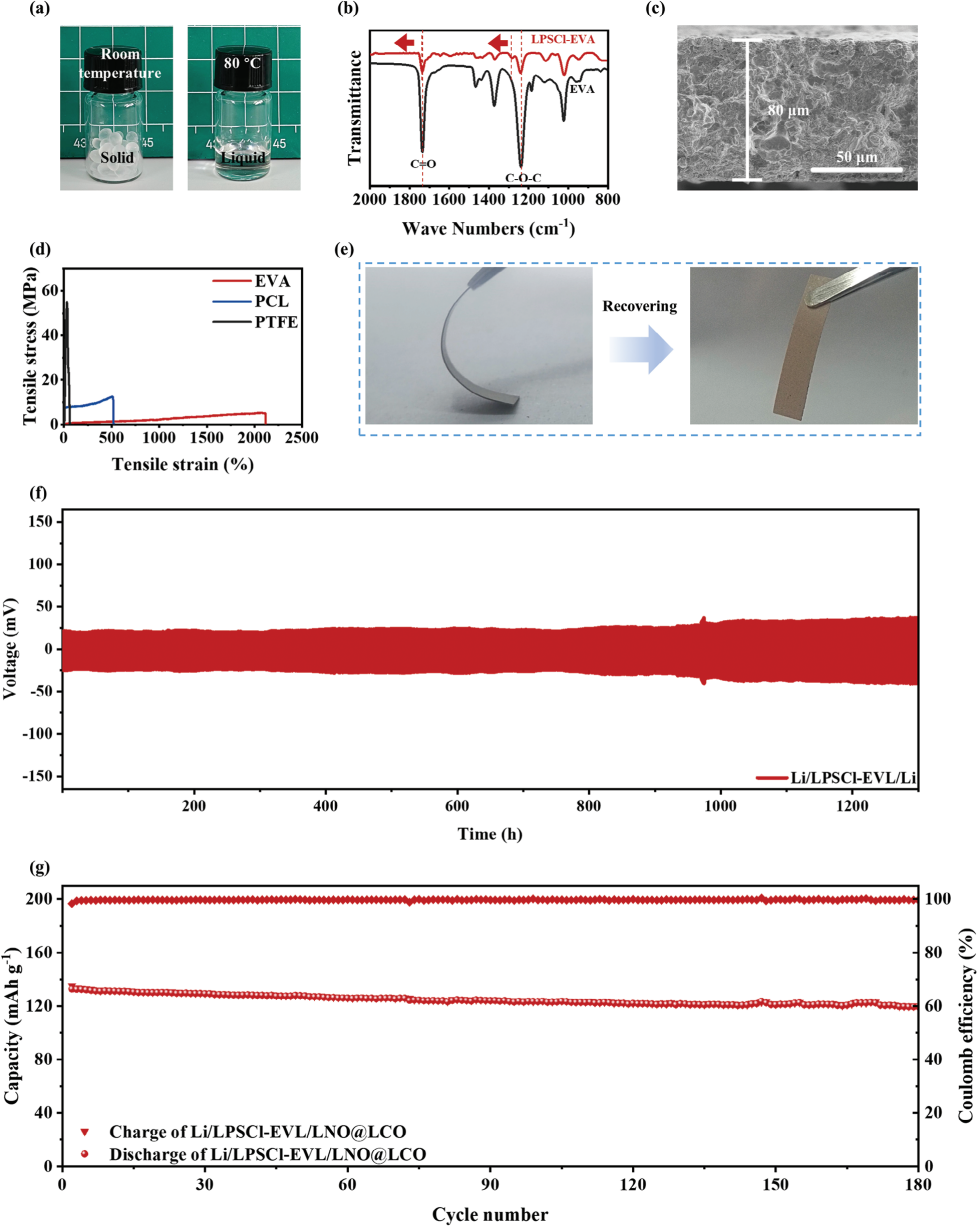
a) Digital images of EVA at room temperature and 80 °C. b) FTIR spectra of EVA and LPSCl‐EVA. c) Cross‐sectional images of LPSCl‐EVL electrolyte. d) Tensile properties of the samples presented in stress‐strain curves. e) Bendability of LPSCl‐EVL electrolyte at room temperature. f) Cycling stability of the Li/Li symmetric cell based on LPSCl‐EVL electrolyte with a current density of 0.3 mA cm^−2^ at the areal capacity of 0.1 mAh cm^−2^. g) Cycling performances of Li/LCO cells assembled with LPSCl‐EVL electrolyte at 0.1 C.

To demonstrate the versatility of the phase transition rationale for binder design to elevate the electrochemical performance of the sulfide electrolytes, we further extend this strategy to a highly flexible thermoplastic elastomer (EVA).^[^ ^42^ ^]^ As mentioned in **Figure** **6**a, EVA can also convert into a liquid at 80 °C. This facilitates the construction of the EVA‐based LPSCl sulfide membrane, which contains densely filled sulfide particles, together with the intermolecular ion‐dipole interaction (Figure 6b,c), collectively promoting the formation of the robust percolating structure. Furthermore, EVA exhibits superior elasticity and breaking strain (2100%), which is 4 times the PCL and 70 times the PTFE (Figure 6d). The intrinsic properties of EVA, characterized by excellent stretchability and elasticity, enable LPSCl membrane based on EVA‐based Li‐conducting binder (LPSCl‐EVL) to present excellent bendability (a bending radius of 4 mm, Figure 6e; Figure S12, Supporting Information), which allows it to accommodate volume changes of Li metal anodes during cycling and effectively mitigate Li dendrite formation at the electrolyte/anode interface. Due to unique interfacial adhesion and the local stress‐dissipation, the Li/Li symmetric cells based on the LPSCl‐EVL membrane exhibit superior cycling stability (1300 h) (Figure 6f). Additionally, the integrated Li/LPSCl‐EVL/LNO@LCO cell also shows excellent long cycling performances with a reversible capacity of 119.6 mAh g^−1^ after 180 cycles (capacity retention of 90.2%) (Figure 6g). This implies that a wider array of polymer binders with different characteristics can be effectively incorporated into high‐performance ASSLBs by leveraging the phase transition strategy, for which sulfide electrolyte particles are dispersed in the liquid state of binders.

## 3 Conclusion

In summary, a phase‐transition strategy is developed to fabricate LPSCl electrolytes leveraging the melted, liquid phase of PLI polymer binders to disperse sulfide electrolyte particles. The PLI binder with phase transition characteristic is susceptible to infiltrating the gaps of LPSCl particles and provides strong interfacial adhesion through the interaction of polar groups in PCL with LPSCl electrolyte, leading to an electrolyte membrane with a thin thickness and high flexibility. Additionally, tailoring the LPSCl with a high‐ion‐conductivity PLI binder facilitates Li‐ion transport in the electrolyte, thus improving the ion conductivity of the sulfide membrane (8.5 × 10^−4^ S cm^−1^). Moreover, the good compatibility of the PLI with the Li metal anode ensures SSE/Li interfacial stability during the whole cycling, contributing to excellent cycle stability of the corresponding Li/Li symmetric cells (1300 h at a current density of 0.3 mA cm^−2^) and Li/LiCoO_2_ full cell (≈90% capacity retention after 300 cycles at 0.1 C). Notably, the “solid‐to‐liquid” strategy can be extended to other polymer binders with phase transition characteristics. The sulfide electrolyte based on EVA binders also exhibits excellent electrochemical performance. The provided insights can be useful for seeking and designing effective binder materials for accessing high‐energy‐density and dendrite‐free ASSLBs.

## 4 Experimental Section

### Materials

LPSCl solid electrolyte powder, Cu and Al foils were purchased from Shenzhen Kejing Material Technology Co., Ltd. PCL (M_w_ = 80 000), EVA (vinyl acetate 40 wt%), Pyr_13_TFSI, paraxylene (PX, ≥99.8%) and acetonitrile (ACN, ≥99.8%) were purchased from Shanghai Macklin Biochemical Co., Ltd. PTFE membranes were purchased from Dongyang Three Microporous Membrane Co., Ltd. PTFE polymer (M_w_ = 10^7^) was purchased from Daikin Fluorochemicals Co., Ltd. LiTFSI and PP (Celgard 2500) membranes were purchased from Dodochem Co., Ltd. Li foils were purchased from Tianjin Zhongneng Lithium Industry Co., Ltd. Vapor grown carbon fiber (VGCF) was purchased from Sigma Aldrich Co., Ltd. LNO@LCO was purchased from Wuhan Solid Li New Energy Technology Co., Ltd. All chemicals were used as received without further purification.

### Synthesis of the Samples

The LPSCl‐PLI membrane was prepared using the dry‐film method. For the PCL‐based Li‐conducting binder, 100 mg PCL, 64 mg LiTFSI, and 30 mg Pyr_13_TFSI ([ester]:[Li^+^] = 4:1) were dissolved in 400 mg ACN under continuous mechanical stirring to obtain a uniform solution. The solution was dried under vacuum at 80 °C for 24 h to obtain a dry Li‐conducting polymer binder. The EVA‐based Li‐conducting binder was prepared by a similar method using PX as a solvent, with a molar ratio of [ester]:[Li^+^] = 6:1. The LPSCl‐PLI membrane was prepared by grinding LPSCl powder and PCL‐based Li‐conducting binder in a weight ratio of 93:7 at 80 °C for 30 min. This mixture was then rolled into a thin film. LPSCl‐EVL (97:3 in weight ratio) and LPSCl‐PTFE membranes (93:7 in weight ratio) membranes were also prepared using the same method. In addition, PCL and LiTFSI with different molar ratios ([ester]:[Li^+^] = 3:1, 4:1, 6:1, 8:1) were also prepared to investigate the influence of lithium salt content on binder ionic conductivity.

### Materials Characterization

The XRD pattern was obtained using a diffractometer (Bruker AXS D8 Advance, Kα radiation, λ = 0.15418 nm). SEM and EDS mapping images were acquired using a SU8010 field‐emission scanning electron microscopy (FESEM). TEM and the corresponding STEM‐EDS images were acquired using a JEOL‐2010F operating at 200 kV. FTIR was conducted using a Thermo Fisher Nicolet iS50. Differential scanning calorimetry (DSC) test was examined by a DSC apparatus (DSC8500) ranging from −20 to 100 at 10 °C min^−1^ in an N_2_ atmosphere. XPS experiment was performed using a PHI‐5400 spectrometer equipped with an Al‐Kα X‐ray source (hν = 1486.6 eV). The mechanical property was characterized on the tensile testing machine (Instron 5969) with a stretching speed of 30 mm min^−1^. The solid‐to‐liquid phase transition strategy of the binder was examined by a polarizing microscope with the hot stage (BX51‐P).

### Electrochemical Measurements

EIS was carried out using a CHI760E electrochemical workstation with an amplitude of 10 mV and a frequency range of 10^6^ to 10^−2^ Hz. The ionic conductivity of the electrolyte was measured by the EIS technique using stainless steel (SS) as the blocking electrode. The Li transference number (t_Li+_) was confirmed by applying a direct current (DC) voltage of ΔV (10 mV) and recording spectra of resistance before and after polarization.

For the symmetric cell test, the CR2025‐type coin cells were assembled in an Ar‐filled glove box (H_2_O < 0.1 ppm and O_2_ < 0.1 ppm). Two pieces of Li foil were attached to both sides of the LPSCl‐PLI or LPSCl‐EVL membranes. No extra stacking pressure was applied during electrochemical testing. The symmetric cells based on LPSCl and LPSCl‐PTFE membranes were assembled under the same process.

The full cells were assembled by LNO@LCO cathodes together with Li metal anodes. The cathode layer is a mixture of LNO@LCO powder, LPSCl powder, VGCF, and PTFE in a weight ratio of 60:34:5:1. This mixture was mixed by manual grinding and then rolled into a thin film by a hot calendar at 80 °C. The obtained LNO@LCO electrode, sulfide electrolyte membrane, and Li foil were pressed together to form an ASSLB. All the galvanostatic charge/discharge cycling measurements were tested on the LAND test system. Full cells were performed at voltages ranging from 2.5 to 4.2 V. The cells were activated at 0.05 C in the first cycle (1 C = 140 mA g^−1^) prior to cycling and then cycled at the current density of 0.1 C.

## Conflict of Interest

The authors declare no conflict of interest.

## Supporting information



Supporting Information

## Data Availability

The data that support the findings of this study are available from the corresponding author upon reasonable request.
