# Supplementary material for: Phase‐Transition‐Promoted Interfacial Anchoring of Sulfide Solid Electrolyte Membranes for High‐Performance All‐Solid‐State Lithium Battery
Source: Adv Sci (Weinh). 2024 Oct 22;11(44):2407798. doi: 10.1002/advs.202407798 (PMC11600274; doi:10.1002/advs.202407798)
Supplement: Supplementary file 1 — Supporting Information [file ADVS-11-2407798-s001.pdf]

ADVANCED SCIENCE
Open Access

Supporting Information

for *Adv. Sci.*, DOI 10.1002/advs.202407798

Phase-Transition-Promoted Interfacial Anchoring of Sulfide Solid Electrolyte Membranes for High-Performance All-Solid-State Lithium Battery

Zhengkang Su, Qinzhe Zhou, Junhong Jin, Shenglin Yang, Guang Li and Jingjing Zhang**

Supporting Information

Phase-Transition-Promoted Interfacial Anchoring of Sulfide Solid Electrolyte Membranes for High-Performance All-Solid-State Lithium Battery

Zhengkang Su, Qinzhe Zhou, Junhong Jin, Shenglin Yang, Guang Li and Jingjing Zhang**

Z. Su, Prof. J. Jin, Prof. S. Yang, Prof. G. Li, Prof. J. Zhang

State Key Laboratory for Modification of Chemical Fibers and Polymer Materials, College of Materials Science and Engineering

Donghua University

Shanghai 201620, P.R. China

E-mail: lig@dhu.edu.cn (G.L.); jjzhang1@dhu.edu.cn (J.Z.)

Q. Zhou

Shanghai Aerospace Power Technology Co., LTD

Shanghai 201112, P.R. China

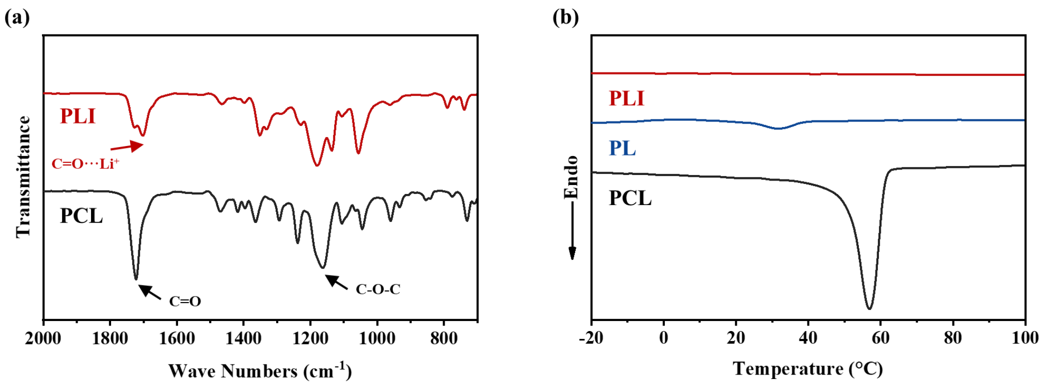

Figure S1. a) FTIR spectra of PCL and PLI. b) DSC profiles of PCL, PL and PLI.

FTIR spectroscopy was employed to clarify the chemical composition and bonding information of PLI. As shown in Figure S1a, PCL exhibits the typical absorbance peaks with the stretching vibration bands of C=O and C-O-C at 1722 and 1162 cm^{-1}, respectively.[1] The peak at 1722 cm^{-1} splits into two peaks in PLI, implying the coordination of the C=O with Li ions for Li-ion conduction.[2] The DSC profiles of PCL, PCL-LiTFSI (denoted as PL) and PLI in Figure S1b show that the melting temperature of PCL decreases significantly after the addition of lithium salt. After further adding ionic liquid, the PLI with the amorphous form shows no sharp melting point, indicating that the degree of crystallinity of PCL is obviously reduced by the plasticizing effect of LiTFSI and $Pyr_{13}TFSI$, thus enhancing the Li^+ transport ability.[3]

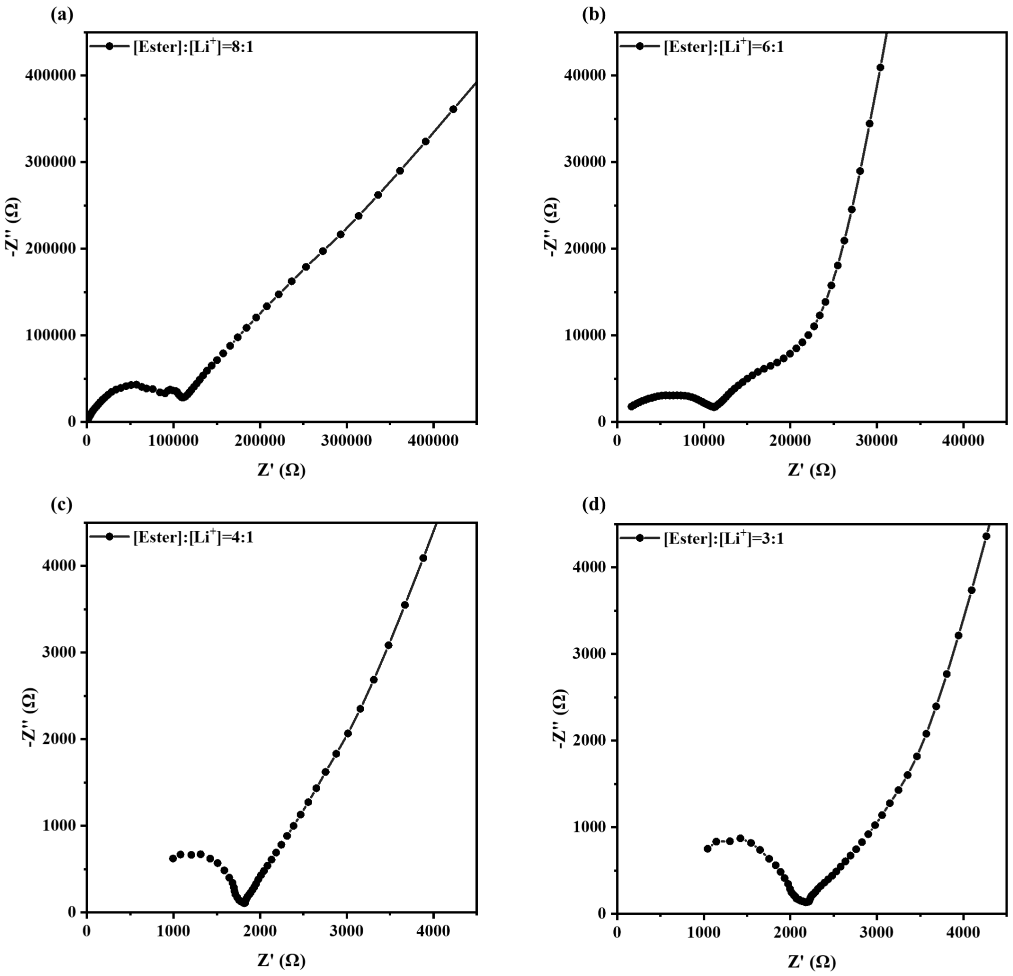

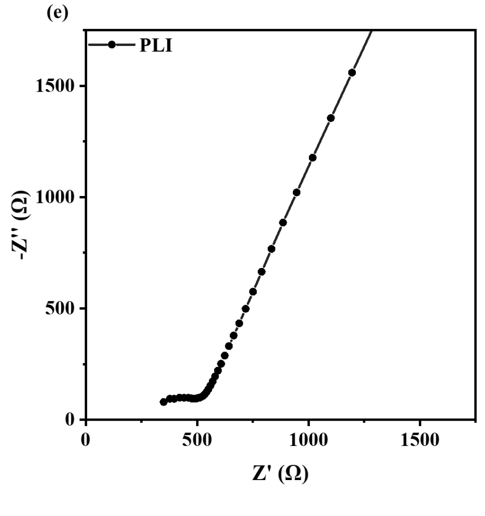

(f)

PCL (wt.%)	LiTFSI (wt.%)	[Ester]: [Li+]	IL (wt.%)	Ionic conductivity (S cm^{-1})
76	24	8:1	0	1.4×10^{-7}
70	30	6:1	0	2.1×10^{-6}
61	39	4:1	0	1.2×10^{-5}
54	46	3:1	0	9.0×10^{-6}
52	33	4:1	15	3.0×10^{-5}

Figure S2. Nyquist plots of a-d) PCL with different LiTFSI content and e) PLI. f) Ionic conductivities of PCL with different LiTFSI content and PLI at room temperature.

The LiTFSI content was varied to optimize the ionic conductivity of PCL, where different [ester]:[Li^+] ratios from 8:1 to 3:1 were prepared. At room temperature, the PCL-LiTFSI with a molar ratio of 4:1 exhibits the highest ionic conductivity (1.2×10^{-5}

S cm^{-1}) (Figure S2a-d,f). Further increase of LiTFSI causes a decrease in ionic conductivity, which is ascribed to the inhibited dissociation of Li^+ from the over-saturated lithium salt.[4] In order to further promote the movement of molecular chains, the 15 wt% Pyr_{13}TFSI was added to the PCL-LiTFSI as a plasticizer,[5] leading to an increased ionic conductivity of 3.0×10^{-5} S cm^{-1} (Figure S2e,f).

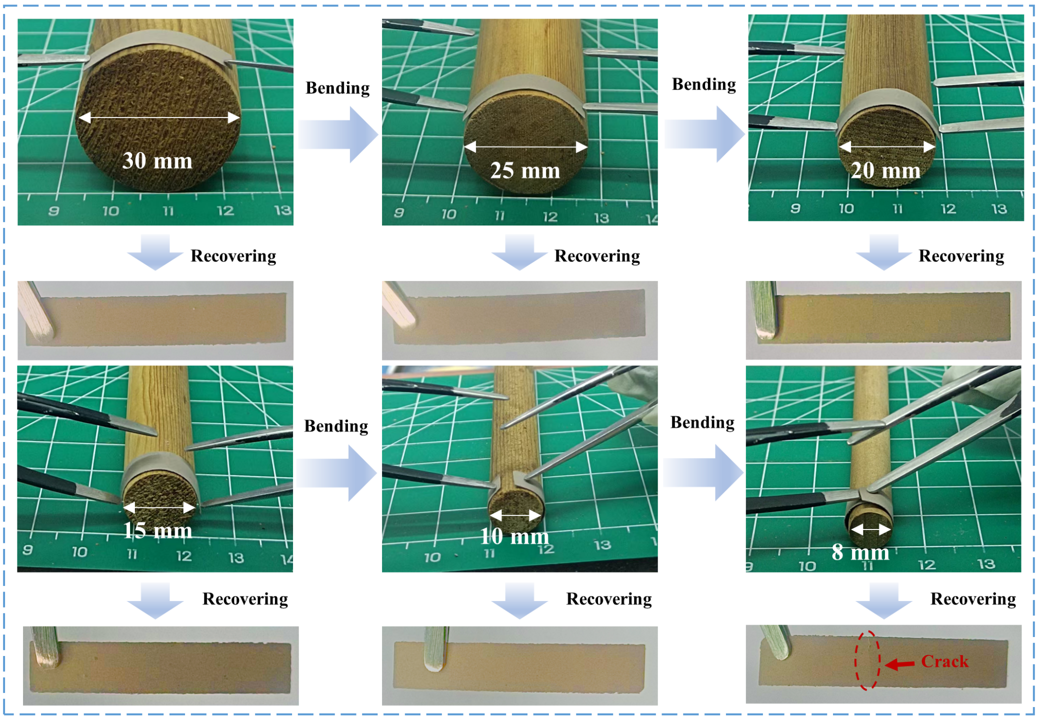

Figure S3. Bending test of LPSCl-PLI membrane using bending rods with various radius.

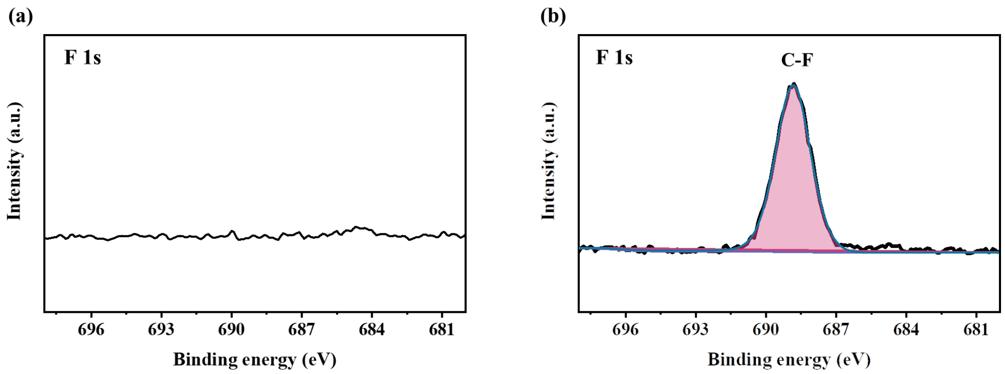

Figure S4. a) F 1s XPS spectra of LPSCl electrolyte. b) F 1s XPS spectra of LPSCl-PLI electrolyte.

(a) (b)

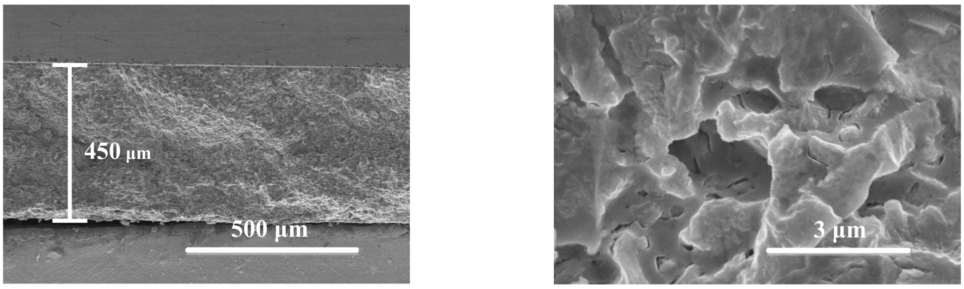

Figure S5. SEM images showing the cross-sectional morphology of the LPSCl membrane fabricated via cold pressing method.

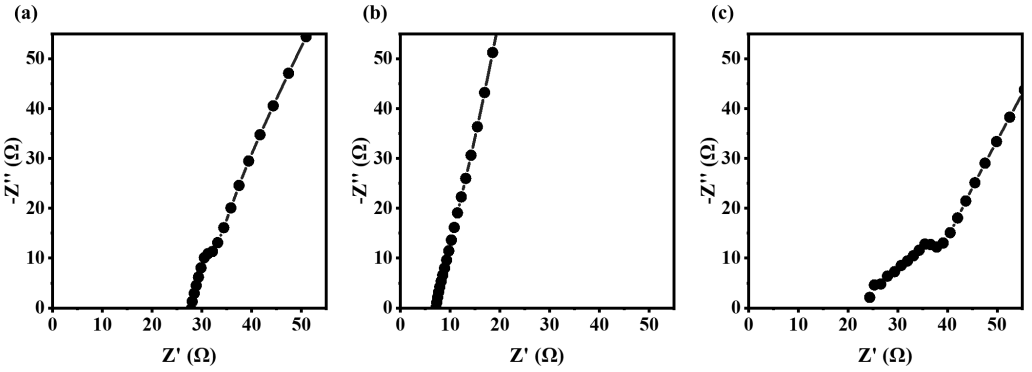

Figure S6. Nyquist plots of a) LPSCl, b) LPSCl-PLI and c) LPSCl-PTFE membranes.

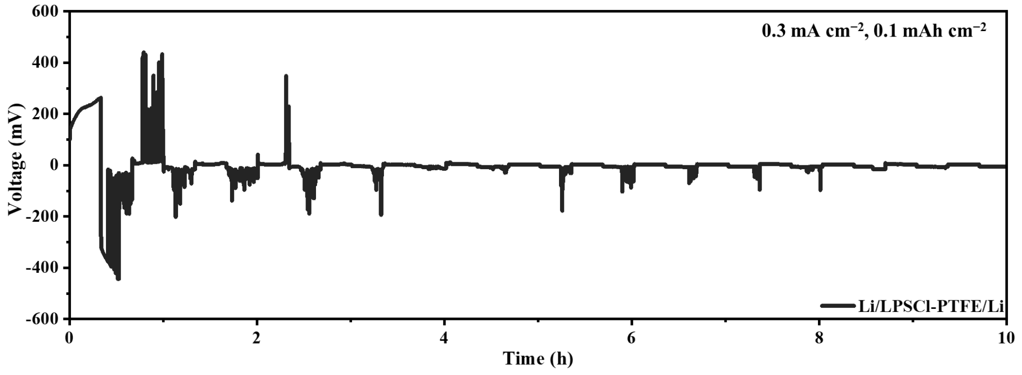

Figure S7. The cycling stability of the Li/Li symmetric cell with LPSCl-PTFE electrolyte with a current density of 0.3 mA cm^{-2} at the areal capacity of 0.1 mAh cm^{-2}.

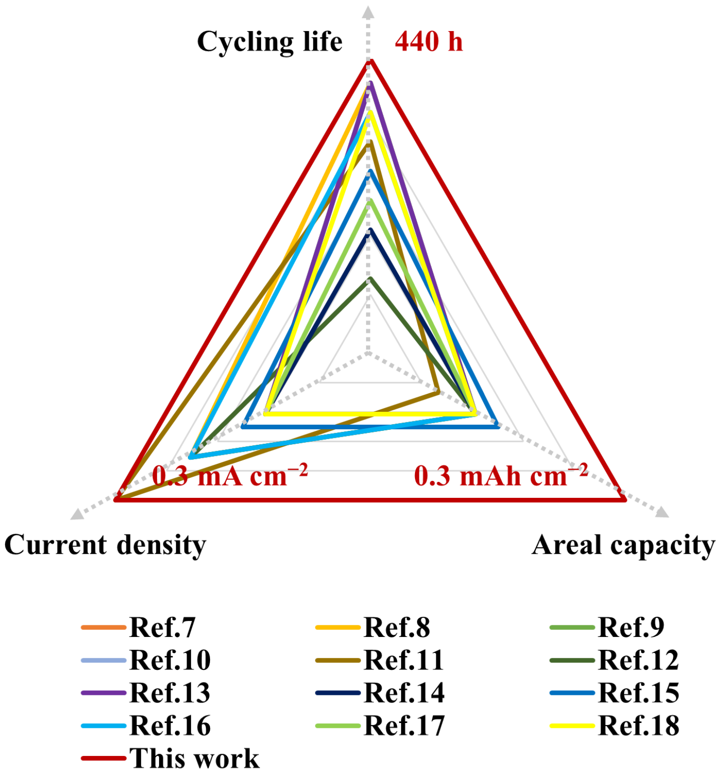

Figure S8. Comparison of the recently reported plating/stripping performances of sulfide SSEs based Li/Li symmetric cells in terms of current density, areal capacity, and cycling life.

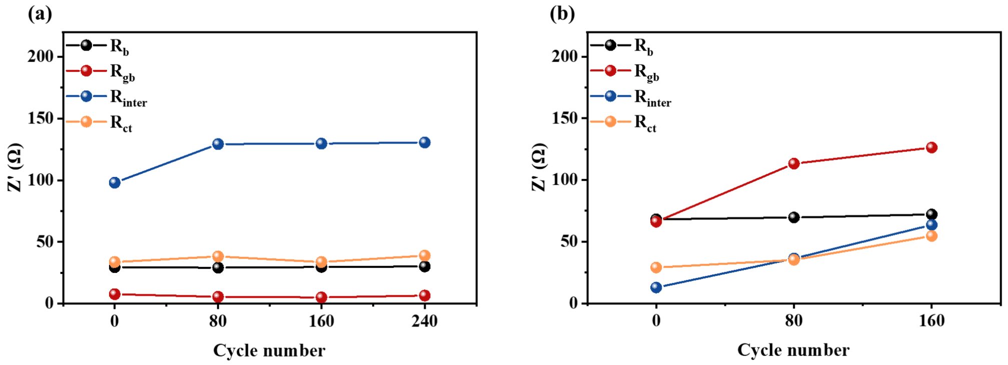

Figure S9. Fitted results of resistance of a) Li/LPSCl-PLI/Li and b) Li/LPSCl/Li cells during cycling.

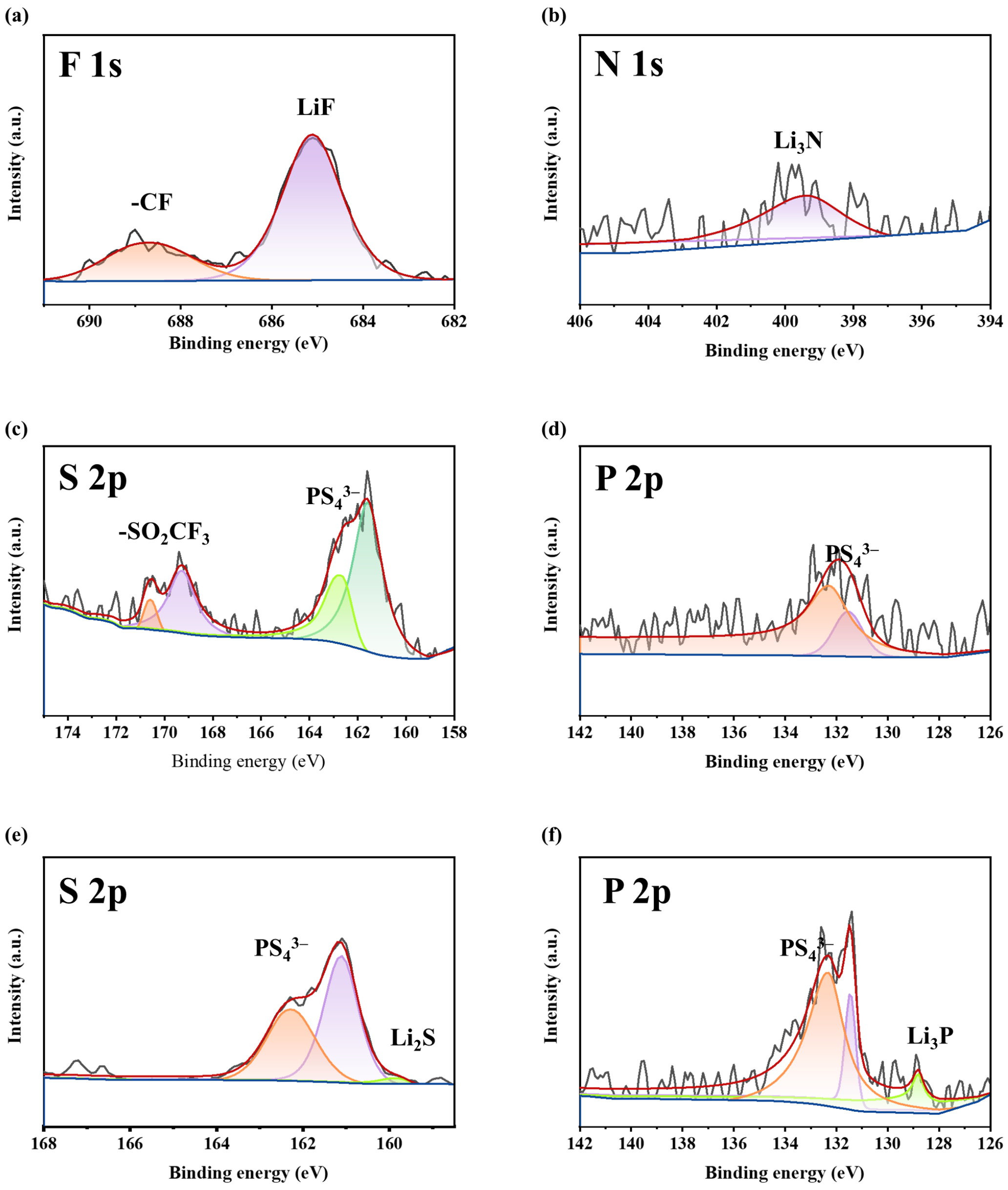

Figure S10. a) F 1s, b) N 1s, c) S 2p and d) P 2p XPS spectra of the cycled Li metal anode from the Li/LPSCl-PLI/Li cell. e) S 2p and f) P 2p XPS spectra of the cycled Li metal anode from the Li/LPSCl/Li cell.

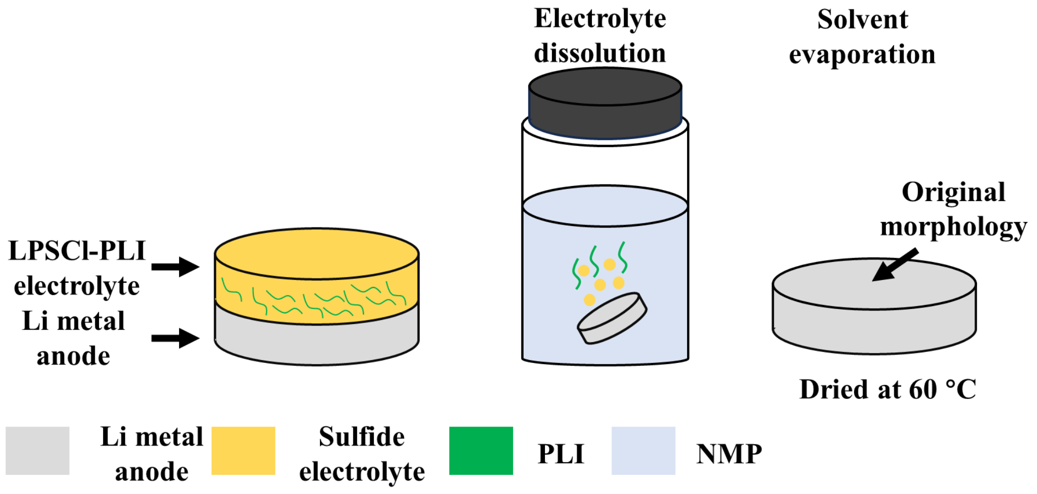

Figure S11. In-situ stripping method for observing the morphology of Li metal anode in sulfide electrolyte based symmetric cells.

N-methyl-2-pyrrolidone (NMP), which has been proven to react with LPSCl and be stable against Li metal, was used to selectively dissolve LPSCl electrolyte without compromising the integrity of Li metal anode. This method facilitates accurate characterization of the surface morphology of Li metal anode after cycling.

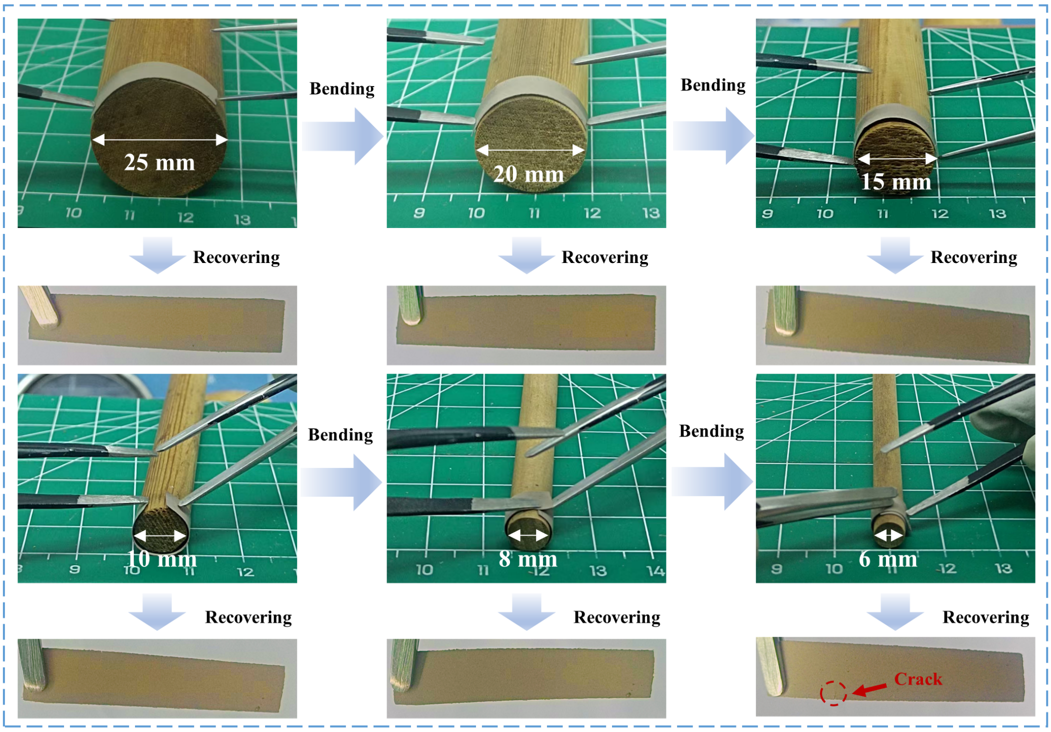

Figure S12. Bending test of LPSCl-EVL membrane using bending rods with various radius.

Table S1. Summary of the plating/stripping performances of Li/Li symmetric cells based on sulfide SSEs in reported literatures.

Cell setup	Sulfide SSEs	Current density (mA cm^{-2})	Li plating/ stripping capacity (mAh cm^{-2})	Cycle time (h)	Reference
Li/Al_2O_3/LPSCl/ Al_2O_3/Li	Li_6PS_5Cl	0.1	0.1	400	[6]
PPC@Li/LPSC/PPC@Li	Li_6PS_5Cl	0.2	0.1	400	[7]
Li/LCE/Li	$Li_{3.25}Ge_{0.25}P_{0.75}S_4$	0.1	0.1	400	[8]
Gel-Li/5PEO-100LGPS-5CTMS+NM/Gel-Li	$Li_{10}GeP_2S_{12}$	0.1	0.1	400	[9]
Li/LPSCl-PEO/Li	Li_6PS_5Cl	0.3	0.05	300	[10]
Li/LPSCl-pvdf-9010/Li	Li_6PS_5Cl	0.2	0.1	67	[11]
Li/PVDF-HFP/PFPEs/20 wt %LGPS/Li	$Li_{10}GeP_2S_{12}$	0.1	0.1	400	[12]
Li/polydopamine-coated Li_6PS_5Cl/Li	Li_6PS_5Cl	0.1	0.1	150	[13]
Li/PCE-LGPS-PCE/Li	$Li_{10}GeP_2S_{12}$	0.13	0.13	250	[14]
Li/PEGDMEL@mix/Li	Li_6PS_5Cl	0.2	0.1	350	[15]
Li/GPE/C-LGPS/GPE/Li	$Li_{10}GeP_2S_{12}$	0.1	0.1	200	[16]
Li/SCSE-4/Li	Li_6PS_5Cl	0.1	0.1	350	[17]
Li/LPSCl-PLI/Li	Li_6PS_5Cl	0.3	0.3	440	This work

Reference

[1] D. Zhang, X. Xu, S. Ji, Z. Wang, Z. Liu, J. Shen, R. Hu, J. Liu, M. Zhu, *ACS Appl. Mater. Interfaces* **2020**, *12*, 21586.

[2] D. Zhang, Z. Liu, Y. Wu, S. Ji, Z. Yuan, J. Liu, M. Zhu, *Adv. Sci.* **2022**, *9*, 2104277.

[3] Y. Seo, Y.-C. Jung, M.-S. Park, D.-W. Kim, *J. Membrane Sci.* **2020**, *603*, 117995.

[4] S. Li, K. Guo, G. Chen, J. Wang, Y. Wang, X. Zhou, Z. Xue, *Energy Storage Mater.* **2022**, *46*, 461.

[5] V. Gregorio, N. Garcia, P. Tiemblo, *Membranes* **2019**, *9*, 50.

[6] Z. Zhang, L. Wu, D. Zhou, W. Weng, X. Yao, *Nano Lett.* **2021**, *21*, 5233.

[7] Y. Chen, W. Li, C. Sun, J. Jin, Q. Wang, X. Chen, W. Zha, Z. Wen, *Adv. Energy Mater.* **2021**, *11*, 2002545.

[8] M. Li, J. E. Frerichs, M. Kolek, W. Sun, D. Zhou, C. J. Huang, B. J. Hwang, M. R. Hansen, M. Winter, P. Bieker, *Adv. Funct. Mater.* **2020**, *30*, 1910123.

[9] H. Liu, P. He, G. Wang, Y. Liang, C. Wang, L.-Z. Fan, *Chem. Eng. J.* **2022**, *430*, 132991.

[10] S. Luo, Z. Wang, A. Fan, X. Liu, H. Wang, W. Ma, L. Zhu, X. Zhang, *J. Power Sources* **2021**, *485*, 229325.

[11] S. Wang, X. Zhang, S. Liu, C. Xin, C. Xue, F. Richter, L. Li, L. Fan, Y. Lin, Y. Shen, J. Janek, C.-W. Nan, *J. Materiomics* **2020**, *6*, 70.

[12] L. Cong, Y. Li, W. Lu, J. Jie, Y. Liu, L. Sun, H. Xie, *J. Power Sources* **2020**, *446*, 227365.

[13] G. Liu, J. Shi, M. Zhu, W. Weng, L. Shen, J. Yang, X. Yao, *Energy Storage Mater.* **2021**, *38*, 249.

[14] C. Wang, K. R. Adair, J. Liang, X. Li, Y. Sun, X. Li, J. Wang, Q. Sun, F. Zhao, X. Lin, R. Li, H. Huang, L. Zhang, R. Yang, S. Lu, X. Sun, *Adv. Funct. Mater.* **2019**, *29*, 1900392.

[15] H. Huo, M. Jiang, B. Mogwitz, J. Sann, Y. Yusim, T.-T. Zuo, Y. Moryson, P. Minnmann, F. H. Richter, C. Veer Singh, J. Janek, *Angew. Chem., Int. Ed.* **2023**, *62*, e202218044.

[16] T. Jiang, P. He, Y. Liang, L.-Z. Fan, *Chem. Eng. J.* **2021**, *421*, 129965.

[17] N. T. Temesgen, H. K. Bezabh, M. A. Weret, K. N. Shitaw, Y. Nikodimos, B. W. Taklu, K. Lakshmanan, S.-C. Yang, S.-K. Jiang, C.-J. Huang, S.-H. Wu, W.-N. Su, B. J. Hwang, *J. Power Sources* **2023**, *556*, 232462.
